# Supplementary material for: Safety and efficacy of intravenous or topical tranexamic acid administration in surgery: a protocol for a systematic review and network meta-analysis
Source: BMJ Open. 2022 May 9;12(5):e058093. doi: 10.1136/bmjopen-2021-058093 (PMC9086642; doi:10.1136/bmjopen-2021-058093)
Supplement: Author's manuscript [file bmjopen-2021-058093.draft_revisions.pdf]

BMJ Open

BMJ Open is committed to open peer review. As part of this commitment we make the peer review history of every article we publish publicly available.

When an article is published we post the peer reviewers' comments and the authors' responses online. We also post the versions of the paper that were used during peer review. These are the versions that the peer review comments apply to.

The versions of the paper that follow are the versions that were submitted during the peer review process. They are not the versions of record or the final published versions. They should not be cited or distributed as the published version of this manuscript.

BMJ Open is an open access journal and the full, final, typeset and author-corrected version of record of the manuscript is available on our site with no access controls, subscription charges or pay-per-view fees (http://bmjopen.bmj.com).

If you have any questions on BMJ Open's open peer review process please email info.bmjopen@bmj.com

BMJ Open

The safety and efficacy of intravenous or topical tranexamic acid administration in surgery: A protocol for systemic review and network meta-analysis

Journal:	*BMJ Open*
Manuscript ID	bmjopen-2021-058093
Article Type:	Protocol
Date Submitted by the Author:	08-Oct-2021

Complete List of Authors:	Wang, Xinyan; Beijing Tiantan Hospital, Anaesthesiology<br>Wang, Xinxin; Beijing Tiantan Hospital, Anaesthesiology<br>Liang, Fa; Beijing Tiantan Hospital, Anesthesiology<br>Yu, Yun; Beijing Tiantan Hospital, Anaesthesiology<br>Han, Ruquan; Beijing Tiantan Hospital,

Keywords:	SURGERY, Blood bank & transfusion medicine < PATHOLOGY, Thromboembolism < CARDIOLOGY

SCHOLARONE™
Manuscripts

The safety and efficacy of intravenous or topical tranexamic acid administration in surgery: A protocol for systemic review and network meta-analysis

Xinyan Wang, B.A; Xinxin Wang, M.D.; Fa Liang, M.D; Yun Yu, M.D.; Ruquan Han, M.D., Ph.D.

Department of Anesthesiology, Beijing Tiantan Hospital, Capital Medical University, Beijing, China

Corresponding author:

Ruquan Han, M.D., Ph.D., Department of Anesthesiology, Beijing Tiantan Hospital, Capital Medical University, Beijing, China

Address: No. 119, Southwest 4th Ring Road, Fengtai District, Beijing, PR China, 100070

Email: ruquan.han@ccmu.edu.cn

Phone (office): 8610-59776660

Email:

Xinyan Wang: alice1428656335@126.com

Xinxin Wang: 635211696@qq.com

Liang Fa: liangfa1001@163.com

Yun Yu: yy_waitou@163.com

Ruquan Han: ruquan.han@gmail.com

Funding: This study was supported by funding from the Ministry of Science and Technology of the People's Republic of China funding (2018YFC2001901) and Clinical Medicine Development of Special Funding Support from the Beijing Municipal Administration of Hospitals (ZYLX201708; DFL20180502).

The authors have no potential conflicts of interest to declare with respect to the research, authorship, and/or publication of this article.

For peer review only

ABSTRACT

Introduction Antifibrinolytic drug tranexamic acid (TXA) has gradually become a hot spot to reduce bleeding in surgery. However, adverse events, such as seizures, pulmonary embolism, and deep vein thrombosis, limited it application. Up to now, far too little attention has been paid to the most optimal dosage and route of tranexamic acid in the field of surgery. Thus, this study uses network meta-analysis method, relying on its characteristics of combining direct comparison and indirect comparison, to analyse the safety and efficacy of different doses (high, medium, low) intravenous injection or of topical application of TXA

Methods and analysis We will search PubMed, Cochrane Central Register of Controlled Trials, Embase, Web of science and China National Knowledge Internet (CNKI) use a strategy that combined the terms tranexamic acid, randomized controlled trials and embolism (or haemorrhage, blood transfusion, seizure, mortality). Two reviewers will independently screen all identified abstracts for eligibility and evaluate the risk of bias of the included studies using the Cochrane risk of bias tool for randomized controlled studies We will conduct a systematic review and network meta-analysis. We plan to investigate this heterogeneity by performing subgroup analyses and sensitivity analysis and we also consider the dose-response relationship of the optimal dose and better routine.

Ethics and dissemination No ethics approval will be sought as no original data will be collected for this review. Findings will be disseminated through peer-reviewed publication and conference presentations.

PROSPERO registration number CRD42021281206

Key words Tranexamic acid; Surgery; Transfusion rate; Thromboembolism

For peer review only

Strengths and limitations of this study

1. The First network meta-analysis focus on optimal dose and better routine of TXA in surgery, not just a single type of surgery
2. The dose-response relationship will be considered in this study and see whether there is a threshold effect.
3. Cochrane risk of bias tool **and** Grading of recommendations assessment, development, and evaluation (GRADE) **are** used for risk of bias assessment and grad**ing** the evidence of all the outcomes**, respectively**.

For peer review only

Background

Bleeding is a major problem in surgery[1, 2]. However, the common treatment—transfusion is limited by few blood sources, high processing costs, adverse blood transfusion reactions, and transmission of infectious diseases, etc[3-5]. Recently, the concept of patient blood management (PBM) has attracted more attention[6, 7]. It has been defined as the timely application of personalised, evidence-based, care bundles of interventions that reduce bleeding and transfusion aiming to improve clinical outcomes[6-8]. For example, autologous blood transfusion, restricted blood transfusion, point-of-care diagnostic test-based algorithms for the personalised treatment of coagulopathy and antifibrinolytic drug[6-8]. Among them, antifibrinolytic drug tranexamic acid (TXA) has gradually become a hot spot.

In 2021, two systematic reviews and meta-analysis of TXA were published in Annal of Surgery[2, 9] The results showed that the perioperative prophylactic topical use or single-dose intravenous tranexamic acid has high safety and low incidence of adverse events[2, 9]. Nonetheless, there are still adverse events, such as seizures, pulmonary embolism, deep vein thrombosis, headache, fatigue, etc[10, 11]. Previous research has established that postoperative seizures are associated with TXA dosage[12], which may be because continuous high concentration of TXA in the brain tissue at the early stage after surgery[13]. Thromboembolic events are another major concern[11, 14], especially in patients which are in a hypercoagulable state due to stress during perioperative period [15, 16]. Tranexamic acid, as an antifibrinolytic drug, tends to increase the coagulation function[17]. Therefore, adopting the appropriate route of administration and dosage has become the primary solution strategy.

The most popular clinical administration of TXA are intravenous infusion and topical use[9, 17, 18]. Up to now, far too little attention has been paid to the most optimal dosage and route of tranexamic acid in the field of surgery[19]. Conducting multi-dose, multiple interventions, large-scale, randomized controlled studies is time-consuming and labour-intensive. Therefore, this study uses network meta-analysis method, relying on its characteristics of combining direct comparison and indirect comparison[20], to analyse the safety and efficacy of different doses (high, medium, low) intravenous injection or of topical application of TXA. We will try to explore the best intervention methods, reduce surgical bleeding, and improve patient outcomes.

For peer review only

Methods and analyses

The review has been registered in PROSPERO International Prospective Register of Systematic reviews (CRD42021281206) (https://www.crd.york.ac.uk/PROSPERO) and will be reported according to Preferred Reporting Items for Systematic reviews and Meta-Analysis protocol (PRISMA-P) guidelines[21]. The PRISMA flow diagram will be used to record every step of the review process **(Figure 1)**. We are planning to start the review in September 2021 and to complete it in December 2022 at the latest.

Sources of evidence and Search Strategy

We will include all published studies fulfilled with criteria without language restriction in this protocol. The published studies are searched in PubMed, Cochrane Central Register of Controlled Trials, Embase, Web of science and China National Knowledge Internet (CNKI) (from the inception dates to September 20th, 2021), (Table 1,2,3,4,5). Unpublished ongoing clinical studies are searched from the World Health Organization International Clinical Trials Registry Platform and ClinicalTrials.gov (until September 20th, 2021). We will continue to update and include the latest articles that meet the search criteria until the deadline. The two researchers (Xinyan Wang and Xinxin Wang) will search separately according to the search strategy ("Tranexamic acid," "Randomized Controlled Trial" and one of the outcomes, specific strategy in Table1,2,3,4,5).

For peer review only

Table 1 Search strategy of Pubmed

Search number	Query
#1	Tranexamic Acid[MeSH Terms]
#2	((((((((((AMCHA[Title/Abstract]) OR (trans-4-(Aminomethyl)cyclohexanecarboxylic Acid[Title/Abstract])) OR (t-AMCHA[Title/Abstract])) OR (AMCA[Title/Abstract])) OR (Anvitoff[Title/Abstract])) OR (Cyklokapron[Title/Abstract])) OR (Ugurol[Title/Abstract])) OR (KABI 2161[Title/Abstract])) OR (Spotof[Title/Abstract])) OR (Transamin[Title/Abstract])) OR (Amchafibrin[Title/Abstract])) OR (Exacyl[Title/Abstract])
#3	#1 or #2
#4	Randomized Controlled Trials as Topic[MeSH Terms]
#5	((((Clinical Trials, Randomized[Title/Abstract]) OR (Trials, Randomized Clinical[Title/Abstract])) OR (Controlled Clinical Trials, Randomized[Title/Abstract])) OR (Clinical trials[Title/Abstract])) OR (Randomized Controlled*[Title/Abstract])
#6	#4 or #5
#7	"Embolism"[MeSH Terms] OR "Embolisms"[Title/Abstract] OR "Embolus"[Title/Abstract] OR "thromboembolism"[MeSH Terms]
#8	"hemorrhage"[MeSH Terms] OR "Hemorrhages"[Title/Abstract] OR "Bleeding"[Title/Abstract]
#9	"blood transfusion"[MeSH Terms] OR "blood transfusion"[MeSH Terms] OR "blood transfusion"[MeSH Terms]
#10	"Seizures"[MeSH Terms] OR "Seizure"[Title/Abstract] OR "Mortality"[MeSH Terms] OR "Mortalities"[Title/Abstract] OR "death rate"[Title/Abstract] OR "case fatality rate"[Title/Abstract] OR "crude mortality rate"[Title/Abstract]
#11	#7 or #8 or #9 or #10
#12	#3 and #6 and #11

For peer review only

Table 2 Search strategy of Cochrane Central Register of Controlled Trials

Search number	Query
#1	MeSH descriptor: [Tranexamic Acid] explode all trees
#2	(AMCHA or t-AMCHA or AMCA or trans-4 Aminomethyl-cyclohexane carboxylic Acid or KABI 2161 or Cyklokapron or Transamin or Spotof or Ugurol or Exacyl or Anvitoff or Amchafibrin):ti,ab,kw (Word variations have been searched)
#3	#1 or #2
#4	MeSH descriptor: [Randomized Controlled Trial] explode all trees
#5	(Randomized Control* or clinical trial*):ti,ab,kw (Word variations have been searched)
#6	#4 or #5
#7	MeSH descriptor: [Hemorrhage] explode all trees
#8	(Bleeding or Hemorrhages):ti,ab,kw (Word variations have been searched)
#9	#7 or #8
#10	MeSH descriptor: [Blood Transfusion] explode all trees
#11	("transfusion"):ti,ab,kw (Word variations have been searched)
#12	#10 or #11
#13	MeSH descriptor: [Mortality] explode all trees
#14	(Mortality Decline or Death Rate or Crude Mortality Rate):ti,ab,kw (Word variations have been searched)
#15	#13 or #14
#16	MeSH descriptor: [Thromboembolism] explode all trees
#17	MeSH descriptor: [Embolism] explode all trees
#18	(Embolisms or Embolus):ti,ab,kw (Word variations have been searched)
#19	MeSH descriptor: [Seizures] explode all trees
#20	#16 or #17 or #18 or #19
#21	#9 or #12 or #15 or #20
#22	#3 and #6 and #21

For peer review only

Table 3 Search strategy of Embase

Search number	Query
#1	'tranexamic acid'/exp
#2	((('amcha'/exp OR amcha OR 't amcha' OR 'amca'/exp OR amca OR 'trans 4') AND 'aminomethyl cyclohexane' AND carboxylic AND ('acid'/exp OR acid) OR kabi) AND 2161 OR 'cyklokapron'/exp OR cyklokapron OR 'transamin'/exp OR transamin OR spotof OR 'ugurol'/exp OR ugurol OR 'exacyl'/exp OR exacyl OR 'anvitoff'/exp OR anvitoff OR 'amchafibrin'/exp OR amchafibrin) AND [abstracts]/lim
#3	#1 OR #2
#4	'randomized controlled trial (topic)'/exp
#5	'clinical trial (topic)'
#6	#4 OR #5
#7	hemorrhage
#8	'bleeding'/exp
#9	#7 OR #8
#10	'blood transfusion'/exp
#11	'transfusion'
#12	#10 OR #11
#13	'mortality'/exp
#14	((mortality AND decline OR death) AND rate OR crude) AND mortality AND rate
#15	#13 OR #14
#16	'thromboembolism'/exp
#17	'embolism'/exp
#18	embolisms OR embolus
#19	#16 OR #17 OR #18
#20	'seizure'/exp
#21	#9 OR #12 OR #15 OR #19 OR #20
#22	#3 AND #6 AND #21

For peer review only

Table 4 Search strategy of Web of Science

Search number	Query
#1	TS=(tranexamic acid)
#2	TS=(AMCHA or (t-AMCHA) or AMCA or (trans-4-(Aminomethyl)cyclohexanecarboxylic Acid) or KABI 2161 or Cyklokapron or Transamin or Spotof or Ugurol or Exacyl or Anvitoff or Amchafibrin)
#3	#1 or #2
#4	TS=(Randomized Controlled Trial)
#5	(TS=(Randomized Control*)) OR TS=(clinical trial*)
#6	#4 or #5
#7	TS=(Hemorrhage) or AB=Bleeding or AB=Hemorrhages
#8	TS=(Blood Transfusion) or AB=transfusion
#9	TS=(Mortality) or AB=Mortality Decline or AB=Death Rate or AB=Crude Mortality Rate
#10	TS=(Thromboembolism) or TS=Embolism or AB=Embolisms or AB=Embolus
#11	TS=(Seizure)
#12	#7 or #8 or #9 or #10 or #11
#13	#3 and #6 and #12

Table 5 Search strategy of China National Knowledge Internet

Search number	Query
#1	检索范围；总库（摘要：氨甲环酸）AND（摘要：随机对照研究）

Inclusion and exclusion criteria

To be included in this systematic review, studies must fulfil each of the criteria outlined below.

Research type (S): randomized controlled trial (RCT).

Participant (P)

Inclusion criteria:

1)Patients (regardless of age) taking surgery, including orthopaedics, neurosurgery, obstetrics and gynaecology, plastic surgery, paediatrics, etc.

2)Intravenous or topical use of TXA, indicating the dosage.

3)Including at least one of the following outcome indicators: blood loss (intraoperative,

For peer review only

postoperative, or total blood loss); blood transfusion; blood transfusion rate; thromboembolic events (deep vein thrombosis, pulmonary embolism), seizures, death

Exclusion criteria: Studies that do not meet the inclusion criteria, such as

1) Inconsistent research types: cohort studies, case-control studies, case reports, reviews, etc.

2) Lack of outcome indicators, lack of odds ratio (OR) and standardized mean difference (SMD) data

Intervention and groups

1) Low topical TXA, ≤1g.

2)Medium doses of topical TXA ,1-2g.

3) high doses of topical TXA, >2g.

4) Intravenous low-dose TXA is infusion dose ≤1g, or initial dose ≤10mg/kg, maintenance dose ≤10mg/kg/h.

5)Intravenous medium-dose TXA: infusion dose 1-2g, or initial dose 10-20mg /kg, maintenance dose⩽15mg /kg/h.

6) Intravenous high-dose TXA: infusion dose ≤1g, or initial dose ＞20mg /kg, maintenance dose ⩽ 20mg /kg/h.

7)Placebo

Outcomes

1)Efficacy outcomes: bleeding volume, blood transfusion volume and blood transfusion rate, operation duration, postoperative Hb and Hct values

2)Safety outcomes: mortality, incidence of thromboembolism and seizures

Languages

No language restriction.

Time

Anticipated start date is September 2021, and anticipated completion date is December 2022.

Study records

Data management

Results of the literature search will be imported into an EndNote X9.3.3 database, and duplicates will be removed. We will establish several independent groups for each selecting stage in the

For peer review only

EndNote database. Abstracts and full-text articles will be uploaded to the database. The extraction information table of final included studies has been designed and the study team will receive training

Selection process

We (Xinyang Wang and Xinxiang Wang) will search studies according to the above search formula, separately. The merged results will be imported into Endnote X9.3.3, and duplicate studies will be removed.

All searched articles will be selected in a two-stage process. First, the title and abstract will be assessed based on the inclusion and exclusion criteria. The study will be removed if it does not meet the criteria. Next, full texts of articles retained in the first round of screening will be retrieved and examined based on eligibility criteria to confirm their inclusion, and studies that do not fulfil the criteria will be removed.

Both steps of the assessment will be performed independently by two reviewers (Xinyang Wang and Xinxiang Wang). If an inconsistency occurs, a third review author resolve conflicts when necessary. We will record reasons for exclusion at both stages of the inclusion process.

Data extraction

We (Xinyang Wang and Xinxiang Wang) will independently extract study type, participants, inclusion criteria, exclusion criteria, baseline characteristics (age, gender, etc.), country, setting, interventions, all outcomes, findings, and study dates from each included study. After data extraction, we will compile the information and import it to excel spreadsheets. When an inconsistency occurs, we will re-check the original document to correct the error. When dealing with missing data, we will contact principal investigators to obtain unreported data or other detailed information.

Risk of bias assessment

We (Xinyang Wang and Xinxiang Wang) will evaluate the risk of bias for each included study independently. The Cochrane risk of bias tool is used to assess randomized controlled studies[22]. The evaluation scale is imported into the Revman software in advance, and specific reasons will be provided for each evaluation characteristic. If an inconsistency occurs, a third review author (Fa

For peer review only

Liang or Yun Yu or Ruquan Han) will be consulted to resolve conflicts.

Statistical analysis

The network meta-analysis was performed by STATA 13.1, Revman 5.3, R software 3.6.0. Risk ratios (RRs) with 95% confidence intervals (CIs) were calculated using the random-effects model for investigating treatment effects. Z test was conducted to assess the significance of overall effect size. A P value of <0.05 was considered statistically significant.

After constructing a heterogeneity matrix, the frequentist method was applied to the fitted meta-regression model. The model covariates as the basic parameters and assumed that heterogeneity is independent of the comparison between effect sizes from multi-arm studies. Inconsistency refers to the differences between direct and various indirect effects estimated for the same comparison. We estimated the probability of a treatment being ranked at a specific place according to the outcome using "network rank."

If evidence suggests moderate statistical or clinical heterogeneity, we plan to investigate this by performing subgroup and sensitivity analyses. We will conduct subgroup and sensitivity analyses based on the actual situation of the included studies. Subgroup analysis will be performed for gender, ethnic group, and different age group, sample sizes etc. since they are very important for dose efficacy.

To explore the specific value of the optimal dose and better routine, the dose-response relationship will be considered in this study and see whether there is a threshold effect.

The publication bias was evaluated by a "comparison-adjusted" funnel plots. GRADEpro software is used to grade the evidence of all the outcomes, and this process is completed by 2 individuals separately.

For peer review only

Contributors: Xinyan Wang: study design, conduct of study, bibliographic research, design of data entry forms, data management, protocol and manuscript writing and review. Xinxin Wang: bibliographic research design and conduct, protocol, and manuscript review. Liang Fa: protocol and manuscript review. Yun Yu: protocol and manuscript review. Ruquan Han: study conception and design, scientific coordination, protocol, and manuscript writing and review.

Funding: This study was supported by funding from Ministry of Science and Technology of the People's Republic of China funding (2018YFC2001901) and Clinical Medicine Development of Special Funding Support from the Beijing Municipal Administration of Hospitals (ZYLX201708; DFL20180502).

Competing interests: None declared.

Patient consent for publication: Not required.

Ethics approval: No ethics approval will be sought as no original data will be collected for this review. Findings will be disseminated through peer-reviewed publication and conference presentations.

Provenance and peer review: Not commissioned; externally peer reviewed.

Open access: This is an open access article distributed in accordance with the Creative Commons Attribution Non Commercial (CC BY-NC 4.0) license, which permits others to distribute, remix, adapt, build upon this work non-commercially, and license their derivative works on different terms, provided the original work is properly cited, appropriate credit is given, any changes made indicated, and the use is non-commercial. See: http://creativecommons.org/licenses/by-nc/4.0/.

For peer review only

REFERENCES

1. Rasouli MR, Maltenfort MG, Erkocak OF ,*et al*. Blood management after total joint arthroplasty in the United States: 19-year trend analysis. *Transfusion* 2016, 56(5):1112-1120.
2. Teoh WY, Tan TG, Ng KT ,*et al*. Prophylactic Topical Tranexamic Acid Versus Placebo in Surgical Patients: A Systematic Review and Meta-analysis*. *Ann Surg* 2021, 273(4):676-683.
3. Alter HJ, Klein HG. The hazards of blood transfusion in historical perspective. *Blood* 2008, 112(7):2617-2626.
4. Spahn DR, Goodnough LT. Alternatives to blood transfusion. *Lancet* 2013, 381(9880):1855-1865.
5. Stokes EA, Wordsworth S, Staves J ,*et al*. Accurate costs of blood transfusion: a microcosting of administering blood products in the United Kingdom National Health Service. *Transfusion* 2018, 58(4):846-853.
6. Spahn DR, Muñoz M, Klein AA ,*et al*. Patient Blood Management: Effectiveness and Future Potential. *Anesthesiology* 2020, 133(1):212-222.
7. Roman MA, Abbasciano RG, Pathak S ,*et al*. Patient blood management interventions do not lead to important clinical benefits or cost-effectiveness for major surgery: a network meta-analysis. *Br J Anaesth* 2021, 126(1):149-156.
8. Mueller MM, Van Remoortel H, Meybohm P ,*et al*. Patient Blood Management: Recommendations From the 2018 Frankfurt Consensus Conference. *Jama* 2019, 321(10):983-997.
9. Heyns M, Knight P, Steve AK ,*et al*. A Single Preoperative Dose of Tranexamic Acid Reduces Perioperative Blood Loss: A Meta-analysis. *Ann Surg* 2021, 273(1):75-81.
10. Chauncey JM, Wieters JS: **Tranexamic Acid**. In: *StatPearls.* edn. Treasure Island (FL): StatPearls Publishing Copyright © 2021, StatPearls Publishing LLC.; 2021.
11. Murao S, Nakata H, Yamakawa K. Safety of tranexamic acid in thrombotic adverse events and seizure in patients with haemorrhage: a protocol for a systematic review and meta-analysis. *BMJ Open* 2020, 10(6):e036020.
12. Kalavrouziotis D, Voisine P, Mohammadi S ,*et al*. High-dose tranexamic acid is an independent predictor of early seizure after cardiopulmonary bypass. *Ann Thorac Surg* 2012, 93(1):148-154.
13. Lecker I, Wang DS, Whissell PD ,*et al*. Tranexamic acid-associated seizures: Causes and treatment. *Ann Neurol* 2016, 79(1):18-26.
14. Poeran J, Chan JJ, Zubizarreta N ,*et al*. Safety of Tranexamic Acid in Hip and Knee Arthroplasty in High-risk Patients. *Anesthesiology* 2021, 135(1):57-68.
15. Wu H, Wang Y, Zhang Y ,*et al*. Breaking the vicious loop between inflammation, oxidative stress and coagulation, a novel anti-thrombus insight of nattokinase by inhibiting LPS-induced inflammation and oxidative stress. *Redox Biol* 2020, 32:101500.
16. Senoner T, Schindler S, Stättner S ,*et al*. Associations of Oxidative Stress and Postoperative Outcome in Liver Surgery with an Outlook to Future Potential Therapeutic Options. *Oxid Med Cell Longev* 2019, 2019:3950818.
17. Xu S, Chen JY, Zheng Q ,*et al*. The safest and most efficacious route of tranexamic acid administration in total joint arthroplasty: A systematic review and network meta-analysis. *Thromb Res* 2019, 176:61-66.
18. Wang X, Yang R, Sun H ,*et al*. Different Effects of Intravenous, Topical, and Combined Application of Tranexamic Acid on Patients with Thoracolumbar Fracture. *World Neurosurg*

For peer review only

2019, 127:e1185-e1189.

19. Fillingham YA, Ramkumar DB, Jevsevar DS *,et al*. The Efficacy of Tranexamic Acid in Total Hip Arthroplasty: A Network Meta-analysis. *J Arthroplasty* 2018, 33(10):3083-3089.e3084.

20. Rouse B, Chaimani A, Li T. Network meta-analysis: an introduction for clinicians. *Intern Emerg Med* 2017, 12(1):103-111.

21. Shamseer L, Moher D, Clarke M *,et al*. Preferred reporting items for systematic review and meta-analysis protocols (PRISMA-P) 2015: elaboration and explanation. *Bmj* 2015, 350:g7647.

22. Higgins JP, Altman DG, Gøtzsche PC *,et al*. The Cochrane Collaboration's tool for assessing risk of bias in randomised trials. *Bmj* 2011, 343:d5928.

For peer review only

Figure and table legends

Table 1. Search Strategy for PubMed

Table 2. Search Strategy for Cochrane Central Register of Controlled Trials

Table 3. Search Strategy for Embase

Table 4. Search Strategy for Web of Science

Table 5. Search Strategy for China National Knowledge Internet

Figure 1. Flow chart diagram presents the selection of articles for systemic review and network meta-analysis of the safety and efficacy of intravenous or topical tranexamic acid administration in surgery.

For peer review only

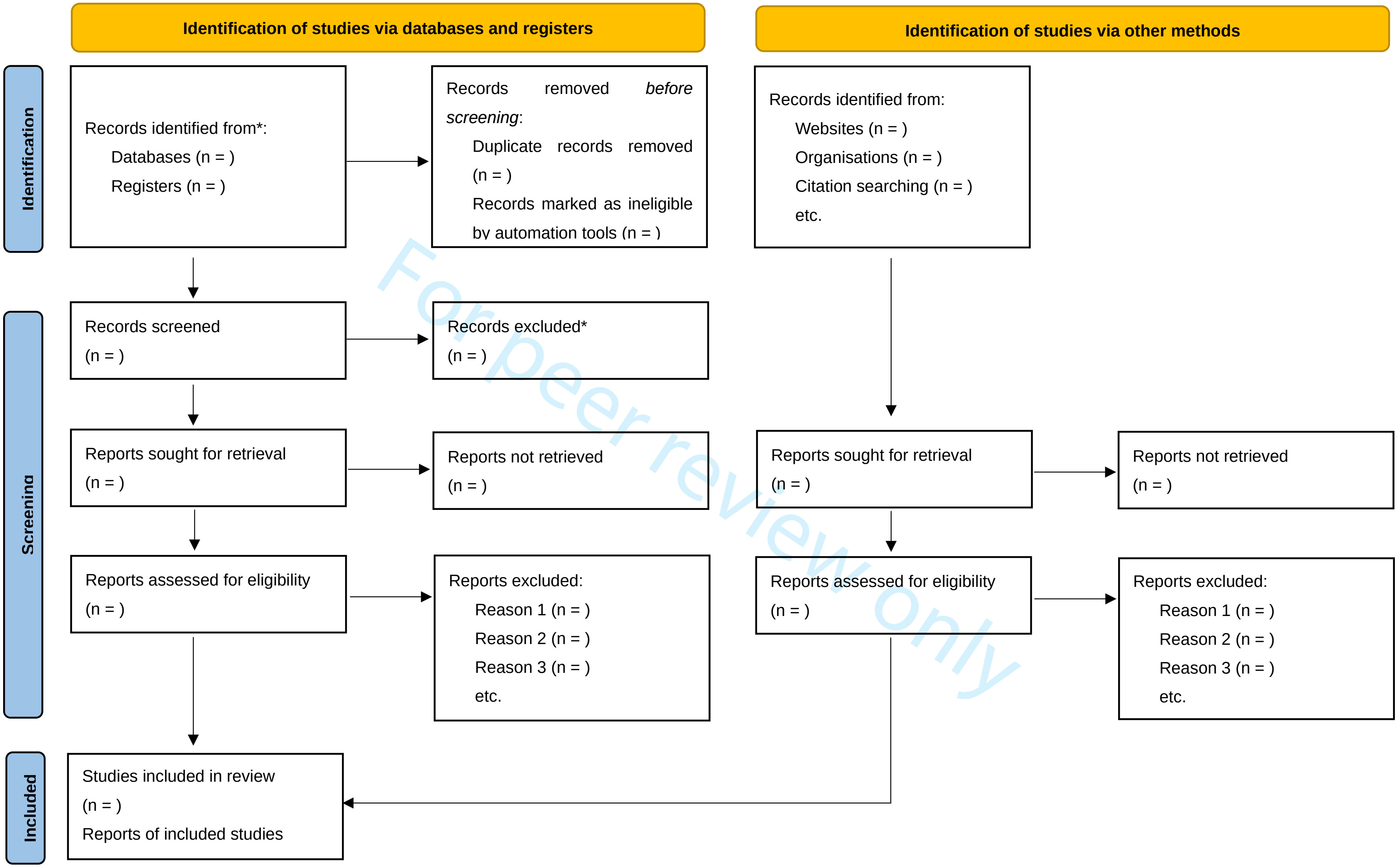

For peer review only

Figure1 PRISMA flow chart. *Consider, if feasible to do so, reporting the number of records identified from each database or register searched (rather than the total number across all databases/registers). **If automation tools were used, indicate how many records were excluded by a human and how many were excluded by automation tools.

PRISMA-P (Preferred Reporting Items for Systematic review and Meta-Analysis Protocols) 2015 checklist: recommended items to address in a systematic review protocol*

Section and topic	Item No	Checklist item	Pages
**ADMINISTRATIVE INFORMATION**			
Title:			
Identification	1a	Identify the report as a protocol of a systematic review	1
Update	1b	If the protocol is for an update of a previous systematic review, identify as such	
Registration	2	If registered, provide the name of the registry (such as PROSPERO) and registration number	2
Authors:			
Contact	3a	Provide name, institutional affiliation, e-mail address of all protocol authors; provide physical mailing address of corresponding author	1
Contributions	3b	Describe contributions of protocol authors and identify the guarantor of the review	1,9
Amendments	4	If the protocol represents an amendment of a previously completed or published protocol, identify as such and list changes; otherwise, state plan for documenting important protocol amendments	
Support:			
Sources	5a	Indicate sources of financial or other support for the review	1,9
Sponsor	5b	Provide name for the review funder and/or sponsor	1,9
Role of sponsor or funder	5c	Describe roles of funder(s), sponsor(s), and/or institution(s), if any, in developing the protocol	1,9
**INTRODUCTION**			
Rationale	6	Describe the rationale for the review in the context of what is already known	4
Objectives	7	Provide an explicit statement of the question(s) the review will address with reference to participants, interventions, comparators, and outcomes (PICO)	4
**METHODS**			
Eligibility criteria	8	Specify the study characteristics (such as PICO, study design, setting, time frame) and report characteristics (such as years considered, language, publication status) to be used as criteria for eligibility for the review	5,6
Information sources	9	Describe all intended information sources (such as electronic databases, contact with study authors, trial registers or other grey literature sources) with planned dates of coverage	5
Search strategy	10	Present draft of search strategy to be used for at least one electronic database, including planned limits, such that it could be repeated	5
Study records:			
Data	11a	Describe the mechanism(s) that will be used to manage records and data throughout the review	6

For peer review only

management			
Selection process	11b	State the process that will be used for selecting studies (such as two independent reviewers) through each phase of the review (that is, screening, eligibility and inclusion in meta-analysis)	6,7
Data collection process	11c	Describe planned method of extracting data from reports (such as piloting forms, done independently, in duplicate), any processes for obtaining and confirming data from investigators	6,7
Data items	12	List and define all variables for which data will be sought (such as PICO items, funding sources), any pre-planned data assumptions and simplifications	6,7
Outcomes and prioritization	13	List and define all outcomes for which data will be sought, including prioritization of main and additional outcomes, with rationale	6
Risk of bias in individual studies	14	Describe anticipated methods for assessing risk of bias of individual studies, including whether this will be done at the outcome or study level, or both; state how this information will be used in data synthesis	7
Data synthesis	15a	Describe criteria under which study data will be quantitatively synthesised	8
	15b	If data are appropriate for quantitative synthesis, describe planned summary measures, methods of handling data and methods of combining data from studies, including any planned exploration of consistency (such as I^2, Kendall's τ)	8
	15c	Describe any proposed additional analyses (such as sensitivity or subgroup analyses, meta-regression)	8
	15d	If quantitative synthesis is not appropriate, describe the type of summary planned	8
Meta-bias(es)	16	Specify any planned assessment of meta-bias(es) (such as publication bias across studies, selective reporting within studies)	8
Confidence in cumulative evidence	17	Describe how the strength of the body of evidence will be assessed (such as GRADE)	8

*** It is strongly recommended that this checklist be read in conjunction with the PRISMA-P Explanation and Elaboration (cite when available) for important clarification on the items. Amendments to a review protocol should be tracked and dated.** **The copyright for PRISMA-P (including checklist) is held by the PRISMA-P Group and is distributed under a Creative Commons Attribution Licence 4.0.**

From: Shamseer L, Moher D, Clarke M, Ghersi D, Liberati A, Petticrew M, Shekelle P, Stewart L, PRISMA-P Group. Preferred reporting items for systematic review and meta-analysis protocols (PRISMA-P) 2015: elaboration and explanation. BMJ. 2015 Jan 2;349(jan02 1):g7647.

For peer review only

BMJ Open

The safety and efficacy of intravenous or topical tranexamic acid administration in surgery: A protocol for systemic review and network meta-analysis

Journal:	*BMJ Open*
Manuscript ID	bmjopen-2021-058093.R1
Article Type:	Protocol
Date Submitted by the Author:	16-Feb-2022
Complete List of Authors:	Wang, Xinyan; Beijing Tiantan Hospital, Anaesthesiology<br>Wang, Xinxin; Beijing Tiantan Hospital, Anaesthesiology<br>Liang, Fa; Beijing Tiantan Hospital, Anesthesiology<br>Yu, Yun; Beijing Tiantan Hospital, Anaesthesiology<br>Han, Ruquan; Beijing Tiantan Hospital,
**Primary Subject Heading**:	Surgery
Secondary Subject Heading:	Surgery
Keywords:	SURGERY, Blood bank & transfusion medicine < PATHOLOGY, Thromboembolism < CARDIOLOGY

SCHOLARONE™
Manuscripts

The safety and efficacy of intravenous or topical tranexamic acid administration in surgery: A protocol for systemic review and network meta-analysis

Xinyan Wang, M.D.; Xinxin Wang, M.D.; Fa Liang, M.D; Yun Yu, M.D.; Ruquan Han, M.D., Ph.D.

Department of Anesthesiology, Beijing Tiantan Hospital, Capital Medical University, Beijing, China

Corresponding author:

Ruquan Han, M.D., Ph.D., Department of Anesthesiology, Beijing Tiantan Hospital, Capital Medical University, Beijing, China

Address: No. 119, Southwest 4th Ring Road, Fengtai District, Beijing, PR China, 100070

Email: ruquan.han@ccmu.edu.cn

Phone (office): 8610-59776660

Email:

Xinyan Wang: alice1428656335@126.com

Xinxin Wang: 635211696@qq.com

Liang Fa: liangfa1001@163.com

Yun Yu: yy_waitou@163.com

Ruquan Han: ruquan.han@gmail.com

Funding: This study was supported by funding from the Clinical Medicine Development of Special Funding Support from the Beijing Municipal Administration of Hospitals (ZYLX201708; DFL20180502).

The authors have no potential conflicts of interest to declare with respect to the research, authorship, and/or publication of this article.

For peer review only

ABSTRACT

Introduction Antifibrinolytic drug tranexamic acid (TXA) has gradually become a hot spot to reduce bleeding in surgery. However, adverse events, such as seizures, pulmonary embolism, and deep vein thrombosis, limited it application. Up to now, far too little attention has been paid to the most optimal dosage and route of tranexamic acid in the field of surgery. Thus, this study uses network meta-analysis method, relying on its characteristics of combining direct comparison and indirect comparison, to analyse the safety and efficacy of different doses (high, medium, low) intravenous injection or of topical application of TXA

Methods and analysis We will search PubMed, Cochrane Central Register of Controlled Trials, Embase, Web of science and China National Knowledge Internet (CNKI) use a strategy that combined the terms tranexamic acid, randomized controlled trials and embolism (or haemorrhage, blood transfusion, seizure, mortality). Two reviewers will independently screen all identified abstracts for eligibility and evaluate the risk of bias of the included studies using the Cochrane risk of bias tool for randomized controlled studies We will conduct a systematic review and network meta-analysis. We plan to investigate this heterogeneity by performing subgroup analysis and sensitivity analysis and we also consider the dose-response relationship of the optimal dose and better routine. Grading of recommendations assessment, development, and evaluation (GRADE) will be used to grade the evidence of all the outcomes.

Ethics and dissemination No ethics approval will be sought as no original data will be collected for this review. Findings will be disseminated through peer-reviewed publication and conference presentations.

PROSPERO registration number CRD42021281206

Key words Tranexamic acid; Surgery; Transfusion rate; Thromboembolism

For peer review only

Strengths and limitations of this study

1. First network meta-analysis focus on optimal dose and better routine of TXA in surgery.
2. Unpublished ongoing clinical studies are searched using the WHO International Clinical Trials Registry Platform and ClinicalTrials.gov.
3. Cochrane risk of bias tool and Newcastle-Ottawa Scale is used for risk of bias assessment.
4. Grading of recommendations assessment, development, and evaluation (GRADE) is used to grade the evidence of all the outcomes.
5. No language restrictions.

For peer review only

Background

Bleeding is a major problem in surgery[1, 2]. However, the common treatment—transfusion is limited by few blood sources, high processing costs, adverse blood transfusion reactions, and transmission of infectious diseases, etc[3-5]. Recently, the concept of patient blood management (PBM) has attracted more attention[6, 7]. It has been defined as the timely application of personalised, evidence-based, care bundles of interventions that reduce bleeding and transfusion aiming to improve clinical outcomes[6-8]. For example, autologous blood transfusion, restricted blood transfusion, point-of-care diagnostic test-based algorithms for the personalised treatment of coagulopathy and antifibrinolytic drug[6-8]. Among them, antifibrinolytic drug tranexamic acid (TXA) has gradually become a hot spot.

In 2021, two systematic reviews and meta-analysis of TXA have published in Annal of Surgery[2, 9] The results showed that the perioperative prophylactic topical use or single-dose intravenous tranexamic acid has high safety and low incidence of adverse events[2, 9]. Nonetheless, there are still adverse events, such as seizures, pulmonary embolism, deep vein thrombosis, headache, fatigue, etc[10, 11]. Previous research has established that postoperative seizures are associated with TXA dosage[12], which may be because continuous high concentration of TXA in the brain tissue at the early stage after surgery[13]. Thromboembolic events are another major concern[11, 14], especially in patients which are in a hypercoagulable state due to stress during perioperative period [15, 16]. Tranexamic acid, as an antifibrinolytic drug, tends to increase the coagulation function[17]. Therefore, adopting the appropriate route of administration and dosage has become the primary solution strategy.

The most popular clinical administration of TXA are intravenous infusion and topical use[9, 17, 18]. Up to now, far too little attention has been paid to the most optimal dosage and route of tranexamic acid in the field of surgery[19]. Conducting multi-dose, multiple interventions, large-scale, randomized controlled studies is time-consuming and labour-intensive. In this network meta-analysis, we plan to analyse the safety and efficacy of different doses (high, medium, low) intravenous injection or of topical application of TXA in surgical patients of all ages, relying on its characteristics of combining direct comparison and indirect comparison[20],

For peer review only

Methods and analyses

The review has been registered in PROSPERO International Prospective Register of Systematic reviews (CRD42021281206) (https://www.crd.york.ac.uk/PROSPERO) and will be reported according to Preferred Reporting Items for Systematic reviews and Meta-Analysis protocol (PRISMA-P) guidelines[21]. The PRISMA flow diagram will be used to record every step of the review process **(Figure 1)**. We are planning to start the review in September 2021 and to complete it in December 2022 at the latest.

Sources of evidence and Search Strategy

We will include all published studies fulfilled with criteria without language restriction in this protocol. The published studies are searched in PubMed, Cochrane Central Register of Controlled Trials, Embase, Web of science and China National Knowledge Internet (CNKI) (from the inception dates to September 20th, 2021), (Table 1, 2, 3, 4, 5). Unpublished ongoing clinical studies are searched from the World Health Organization International Clinical Trials Registry Platform and ClinicalTrials.gov (until September 20th, 2021). We will continue to update and include the latest articles that meet the search criteria until the deadline. The two researchers (Xinyan Wang and Xinxin Wang) will search separately according to the search strategy ("Tranexamic acid", " Randomized Controlled Trial " and one of the outcomes, specific strategy in Table1,2,3,4,5).

For peer review only

Table 1 Search strategy of PubMed

Search number	Query
#1	Tranexamic Acid [MeSH Terms]
#2	((((((((((AMCHA[Title/Abstract]) OR (trans-4-(Aminomethyl)cyclohexanecarboxylic Acid[Title/Abstract])) OR (t-AMCHA[Title/Abstract])) OR (AMCA[Title/Abstract])) OR (Anvitoff[Title/Abstract])) OR (Cyklokapron[Title/Abstract])) OR (Ugurol[Title/Abstract])) OR (KABI 2161[Title/Abstract])) OR (Spotof[Title/Abstract])) OR (Transamin[Title/Abstract])) OR (Amchafibrin[Title/Abstract])) OR (Exacyl[Title/Abstract])
#3	#1 or #2
#4	Randomized Controlled Trials as Topic [MeSH Terms]
#5	((((Clinical Trials, Randomized [Title/Abstract]) OR (Trials, Randomized Clinical [Title/Abstract])) OR (Controlled Clinical Trials, Randomized [Title/Abstract])) OR (Clinical trials [Title/Abstract])) OR (Randomized Controlled*[Title/Abstract])
#6	#4 or #5
#7	"Embolism"[MeSH Terms] OR "Embolisms"[Title/Abstract] OR "Embolus"[Title/Abstract] OR "thromboembolism"[MeSH Terms] OR "Thrombosis"[Mesh] OR "Venous Thrombosis"[Mesh]
#8	"hemorrhage"[MeSH Terms] OR "Hemorrhages"[Title/Abstract] OR "Bleeding"[Title/Abstract]
#9	"Blood transfusion"[MeSH Terms] OR "blood transfusion"[MeSH Terms] OR "blood transfusion"[MeSH Terms]
#10	"Seizures"[MeSH Terms] OR "Seizure"[Title/Abstract] OR "Mortality"[MeSH Terms] OR "Mortalities"[Title/Abstract] OR "death rate"[Title/Abstract] OR "case fatality rate"[Title/Abstract] OR "crude mortality rate"[Title/Abstract]
#11	#7 or #8 or #9 or #10
#12	#3 and #6 and #11

For peer review only

Table 2 Search strategy of Cochrane Central Register of Controlled Trials

Search number	Query
#1	MeSH descriptor: [Tranexamic Acid] explode all trees
#2	(AMCHA or t-AMCHA or AMCA or trans-4 Aminomethyl-cyclohexane carboxylic Acid or KABI 2161 or Cyklokapron or Transamin or Spotof or Ugurol or Exacyl or Anvitoff or Amchafibrin): ti, ab, kw (Word variations have been searched)
#3	#1 or #2
#4	MeSH descriptor: [Randomized Controlled Trial] explode all trees
#5	(Randomized Control* or clinical trial*): ti, ab, kw (Word variations have been searched)
#6	#4 or #5
#7	MeSH descriptor: [Hemorrhage] explode all trees
#8	(Bleeding or Hemorrhages): ti, ab, kw (Word variations have been searched)
#9	#7 or #8
#10	MeSH descriptor: [Blood Transfusion] explode all trees
#11	("transfusion"): ti, ab, kw (Word variations have been searched)
#12	#10 or #11
#13	MeSH descriptor: [Mortality] explode all trees
#14	(Mortality Decline or Death Rate or Crude Mortality Rate): ti, ab, kw (Word variations have been searched)
#15	#13 or #14
#16	MeSH descriptor: [Thromboembolism] explode all trees
#17	MeSH descriptor: [Embolism] explode all trees
#18	(Embolisms or Embolus): ti, ab, kw (Word variations have been searched)
#19	MeSH descriptor: [Thrombosis] explode all trees
#20	MeSH descriptor: [Venous Thrombosis] explode all trees
#21	MeSH descriptor: [Seizures] explode all trees
#22	#16 or #17 or #18 or #19 or #20 or #21
#23	#9 or #12 or #15 or #22
#24	#3 and #6 and #23

For peer review only

Table 3 Search strategy of Embase

Search number	Query
#1	'Tranexamic acid'/exp
#2	((('amcha'/exp OR amcha OR 't amcha' OR 'amca'/exp OR amca OR 'trans 4') AND 'aminomethyl cyclohexane' AND carboxylic AND ('acid'/exp OR acid) OR kabi) AND 2161 OR 'cyklokapron'/exp OR cyklokapron OR 'transamin'/exp OR transamin OR spotof OR 'ugurol'/exp OR ugurol OR 'exacyl'/exp OR exacyl OR 'anvitoff'/exp OR anvitoff OR 'amchafibrin'/exp OR amchafibrin) AND [abstracts]/lim
#3	#1 OR #2
#4	'Randomized controlled trial (topic)'/exp
#5	'Clinical trial (topic)'
#6	#4 OR #5
#7	Hemorrhage
#8	'bleeding'/exp
#9	#7 OR #8
#10	'Blood transfusion'/exp
#11	'transfusion'
#12	#10 OR #11
#13	'mortality'/exp
#14	((mortality AND decline OR death) AND rate OR crude) AND mortality AND rate
#15	#13 OR #14
#16	'thromboembolism'/exp
#17	'embolism'/exp
#18	embolisms OR embolus
#19	'thrombosis'/exp
#20	'Venous thrombosis'/exp
#21	#16 OR #17 OR #18 OR #19 OR #20
#22	'seizure'/exp
#23	#9 OR #12 OR #15 OR #21 OR #23
#24	#3 AND #6 AND #23

For peer review only

Table 4 Search strategy of Web of Science

Search number	Query
#1	TS= (tranexamic acid)
#2	TS= (AMCHA or (t-AMCHA) or AMCA or (trans-4-(Aminomethyl) cyclohexanecarboxylic Acid) or KABI 2161 or Cyklokapron or Transamin or Spotof or Ugurol or Exacyl or Anvitoff or Amchafibrin)
#3	#1 or #2
#4	TS= (Randomized Controlled Trial)
#5	(TS= (Randomized Control*)) OR TS= (clinical trial*)
#6	#4 or #5
#7	TS=(Hemorrhage) or AB=Bleeding or AB=Hemorrhages
#8	TS= (Blood Transfusion) or AB=transfusion
#9	TS=(Mortality) or AB=Mortality Decline or AB=Death Rate or AB=Crude Mortality Rate
#10	TS=(Thromboembolism) or TS=Embolism or AB=Embolisms or AB=Embolus
#11	TS=(Thrombosis) or TS= (Venous thrombosis)
#12	TS=(Seizure)
#13	#7 or #8 or #9 or #10 or #11 or #12
#14	#3 and #6 and #13

Table 5 Search strategy of China National Knowledge Internet

Search number	Query
#1	检索范围；总库（摘要：氨甲环酸）AND（摘要：随机对照研究）

Inclusion and exclusion criteria

To be included in this systematic review, studies must fulfil each of the criteria outlined below.

Research type (S): randomized controlled trial (RCT).

Participant (P)

Inclusion criteria:

1)Patients (regardless of age) taking surgery, including orthopaedics, neurosurgery, obstetrics and gynaecology, plastic surgery, paediatrics, etc.

For peer review only

2)Intravenous or topical use of TXA, indicating the dosage.

3)Including at least one of the following outcome indicators: blood loss (intraoperative, postoperative, or total blood loss); blood transfusion; blood transfusion rate; thromboembolic events (deep vein thrombosis, pulmonary embolism), seizures, death

Exclusion criteria: Studies that do not meet the inclusion criteria, such as

1) Inconsistent research types: cohort studies, case-control studies, case reports, reviews, etc.

2) Lack of outcome indicators, lack of odds ratio (OR) and standardized mean difference (SMD) data

Intervention and groups

1) Low topical TXA, ≤1g.

2)Medium doses of topical TXA ,1-2g.

3) high doses of topical TXA, ＞2g.

4) Intravenous low-dose TXA is infusion dose ≤1g, or initial dose ≤10mg/kg, maintenance dose ≤10mg/kg/h.

5)Intravenous medium-dose TXA: infusion dose 1-2g, or initial dose 10-20mg /kg, maintenance dose⩽15mg /kg/h.

6) Intravenous high-dose TXA: infusion dose ＞2g, or initial dose ＞20mg /kg, maintenance dose ⩽20mg /kg/h.

7)Placebo

Outcomes

1)Efficacy outcomes: bleeding volume, blood transfusion volume and blood transfusion rate, operation duration, postoperative Hb and Hct values, change in Hb or Hct, and drain output.

2)Safety outcomes: mortality, incidence of thromboembolism, seizures and hematoma.

Languages

No language restriction.

Time

Anticipated start date is September 2021, and anticipated completion date is December 2022.

Study records

Data management

For peer review only

Results of the literature search will be imported into an EndNote X9.3.3 database, and duplicates will be removed. We will establish several independent groups for each selecting stage in the EndNote database. Abstracts and full-text articles will be uploaded to the database. The extraction information table of final included studies has been designed and the study team will receive training

Selection process

We (Xinyang Wang and Xinxiang Wang) will search studies according to the above search formula, separately. The merged results will be imported into Endnote X9.3.3, and duplicate studies will be removed.

All searched articles will be selected in a two-stage process. First, the title and abstract will be assessed based on the inclusion and exclusion criteria. The study will be removed if it does not meet the criteria. Next, full texts of articles retained in the first round of screening will be retrieved and examined based on eligibility criteria to confirm their inclusion, and studies that do not fulfil the criteria will be removed.

Both steps of the assessment will be performed independently by two reviewers (Xinyang Wang and Xinxiang Wang). If an inconsistency occurs, a third review author resolve conflicts when necessary. We will record reasons for exclusion at both stages of the inclusion process.

Data extraction

We (Xinyang Wang and Xinxiang Wang) will independently extract study type, participants, inclusion criteria, exclusion criteria, baseline characteristics (age, gender, etc.), country, setting, interventions, all outcomes, findings, and study dates from each included study. After data extraction, we will compile the information and import it to excel spreadsheets. When an inconsistency occurs, we will re-check the original document to correct the error. When dealing with missing data, we will contact principal investigators to obtain unreported data or other detailed information.

Risk of bias assessment

We (Xinyang Wang and Xinxiang Wang) will evaluate the risk of bias for each included study

For peer review only

independently. The Cochrane risk of bias tool is used to assess randomized controlled studies[22]. The evaluation scale is imported into the Revman software in advance, and specific reasons will be provided for each evaluation characteristic. If an inconsistency occurs, a third review author (Fa Liang or Yun Yu or Ruquan Han) will be consulted to resolve conflicts.

Statistical analysis

The network meta-analysis was performed by STATA 13.1, Revman 5.3, R software 3.6.0. Risk ratios (RRs) with 95% confidence intervals (CIs) were calculated using the random-effects model for investigating treatment effects. Z test was conducted to assess the significance of overall effect size. A P value of <0.05 was considered statistically significant.

After constructing a heterogeneity matrix, the frequentist method was applied to the fitted meta-regression model. The model covariates as the basic parameters and assumed that heterogeneity is independent of the comparison between effect sizes from multi-arm studies. Inconsistency refers to the differences between direct and various indirect effects estimated for the same comparison. We estimated the probability of a treatment being ranked at a specific place according to the outcome using "network rank."

If evidence suggests moderate statistical or clinical heterogeneity, we plan to investigate this by performing subgroup and sensitivity analyses. We will conduct subgroup and sensitivity analyses based on the actual situation of the included studies. Subgroup analysis will be performed for gender, ethnic group, and different age group, sample sizes, type of surgery, etc., since they are particularly important for dose efficacy. Sensitivity analysis will be planned without patients undergoing cardiac surgery and without pediatric patients.

To explore the specific value of the optimal dose and better routine, the dose-response relationship will be considered in this study and see whether there is a threshold effect.

The publication bias was evaluated by a "comparison-adjusted" funnel plot. GRADEpro software is used to grade the evidence of all the outcomes, and this process is completed by two individuals separately.

For peer review only

Contributors: Xinyan Wang: study design, conduct of study, bibliographic research, design of data entry forms, data management, protocol and manuscript writing and review. Xinxin Wang: bibliographic research design and conduct, protocol, and manuscript review. Liang Fa: protocol and manuscript review. Yun Yu: protocol and manuscript review. Ruquan Han: study conception and design, scientific coordination, protocol, and manuscript writing and review.

Funding: This study was supported by funding from the Clinical Medicine Development of Special Funding Support from the Beijing Municipal Administration of Hospitals (ZYLX201708; DFL20180502).

Competing interests: None declared.

Patient consent for publication: Not required.

Ethics approval: No ethics approval will be sought as no original data will be collected for this review. Findings will be disseminated through peer-reviewed publication and conference presentations.

Provenance and peer review: Not commissioned; externally peer reviewed.

Open access: This is an open access article distributed in accordance with the Creative Commons Attribution Non-Commercial (CC BY-NC 4.0) license, which permits others to distribute, remix, adapt, build upon this work non-commercially, and license their derivative works on different terms, provided the original work is properly cited, appropriate credit is given, any changes made indicated, and the use is non-commercial. See: http://creativecommons.org/licenses/by-nc/4.0/.

For peer review only

REFERENCES

1. Rasouli MR, Maltenfort MG, Erkocak OF ,*et al*. Blood management after total joint arthroplasty in the United States: 19-year trend analysis. *Transfusion* 2016, 56(5):1112-1120.
2. Teoh WY, Tan TG, Ng KT ,*et al*. Prophylactic Topical Tranexamic Acid Versus Placebo in Surgical Patients: A Systematic Review and Meta-analysis*. *Ann Surg* 2021, 273(4):676-683.
3. Alter HJ, Klein HG. The hazards of blood transfusion in historical perspective. *Blood* 2008, 112(7):2617-2626.
4. Spahn DR, Goodnough LT. Alternatives to blood transfusion. *Lancet* 2013, 381(9880):1855-1865.
5. Stokes EA, Wordsworth S, Staves J ,*et al*. Accurate costs of blood transfusion: a microcosting of administering blood products in the United Kingdom National Health Service. *Transfusion* 2018, 58(4):846-853.
6. Spahn DR, Muñoz M, Klein AA ,*et al*. Patient Blood Management: Effectiveness and Future Potential. *Anesthesiology* 2020, 133(1):212-222.
7. Roman MA, Abbasciano RG, Pathak S ,*et al*. Patient blood management interventions do not lead to important clinical benefits or cost-effectiveness for major surgery: a network meta-analysis. *Br J Anaesth* 2021, 126(1):149-156.
8. Mueller MM, Van Remoortel H, Meybohm P ,*et al*. Patient Blood Management: Recommendations From the 2018 Frankfurt Consensus Conference. *Jama* 2019, 321(10):983-997.
9. Heyns M, Knight P, Steve AK ,*et al*. A Single Preoperative Dose of Tranexamic Acid Reduces Perioperative Blood Loss: A Meta-analysis. *Ann Surg* 2021, 273(1):75-81.
10. Chauncey JM, Wieters JS: **Tranexamic Acid**. In: *StatPearls.* edn. Treasure Island (FL): StatPearls Publishing Copyright © 2021, StatPearls Publishing LLC.; 2021.
11. Murao S, Nakata H, Yamakawa K. Safety of tranexamic acid in thrombotic adverse events and seizure in patients with haemorrhage: a protocol for a systematic review and meta-analysis. *BMJ Open* 2020, 10(6):e036020.
12. Kalavrouziotis D, Voisine P, Mohammadi S ,*et al*. High-dose tranexamic acid is an independent predictor of early seizure after cardiopulmonary bypass. *Ann Thorac Surg* 2012, 93(1):148-154.
13. Lecker I, Wang DS, Whissell PD ,*et al*. Tranexamic acid-associated seizures: Causes and treatment. *Ann Neurol* 2016, 79(1):18-26.
14. Poeran J, Chan JJ, Zubizarreta N ,*et al*. Safety of Tranexamic Acid in Hip and Knee Arthroplasty in High-risk Patients. *Anesthesiology* 2021, 135(1):57-68.
15. Wu H, Wang Y, Zhang Y ,*et al*. Breaking the vicious loop between inflammation, oxidative stress and coagulation, a novel anti-thrombus insight of nattokinase by inhibiting LPS-induced inflammation and oxidative stress. *Redox Biol* 2020, 32:101500.
16. Senoner T, Schindler S, Stättner S ,*et al*. Associations of Oxidative Stress and Postoperative Outcome in Liver Surgery with an Outlook to Future Potential Therapeutic Options. *Oxid Med Cell Longev* 2019, 2019:3950818.
17. Xu S, Chen JY, Zheng Q ,*et al*. The safest and most efficacious route of tranexamic

For peer review only

acid administration in total joint arthroplasty: A systematic review and network meta-analysis. *Thromb Res* 2019, 176:61-66.

18. Wang X, Yang R, Sun H ,*et al*. Different Effects of Intravenous, Topical, and Combined Application of Tranexamic Acid on Patients with Thoracolumbar Fracture. *World Neurosurg* 2019, 127:e1185-e1189.
19. Fillingham YA, Ramkumar DB, Jevsevar DS ,*et al*. The Efficacy of Tranexamic Acid in Total Hip Arthroplasty: A Network Meta-analysis. *J Arthroplasty* 2018, 33(10):3083-3089.e3084.
20. Rouse B, Chaimani A, Li T. Network meta-analysis: an introduction for clinicians. *Intern Emerg Med* 2017, 12(1):103-111.
21. Shamseer L, Moher D, Clarke M ,*et al*. Preferred reporting items for systematic review and meta-analysis protocols (PRISMA-P) 2015: elaboration and explanation. *Bmj* 2015, 350:g7647.
22. Higgins JP, Altman DG, Gøtzsche PC ,*et al*. The Cochrane Collaboration's tool for assessing risk of bias in randomised trials. *Bmj* 2011, 343:d5928.

For peer review only

Figure and table legends

Table 1. Search Strategy for PubMed

Table 2. Search Strategy for Cochrane Central Register of Controlled Trials

Table 3. Search Strategy for Embase

Table 4. Search Strategy for Web of Science

Table 5. Search Strategy for China National Knowledge Internet

Figure 1. Flow chart diagram presents the selection of articles for systemic review and network meta-analysis of the safety and efficacy of intravenous or topical tranexamic acid administration in surgery.

For peer review only

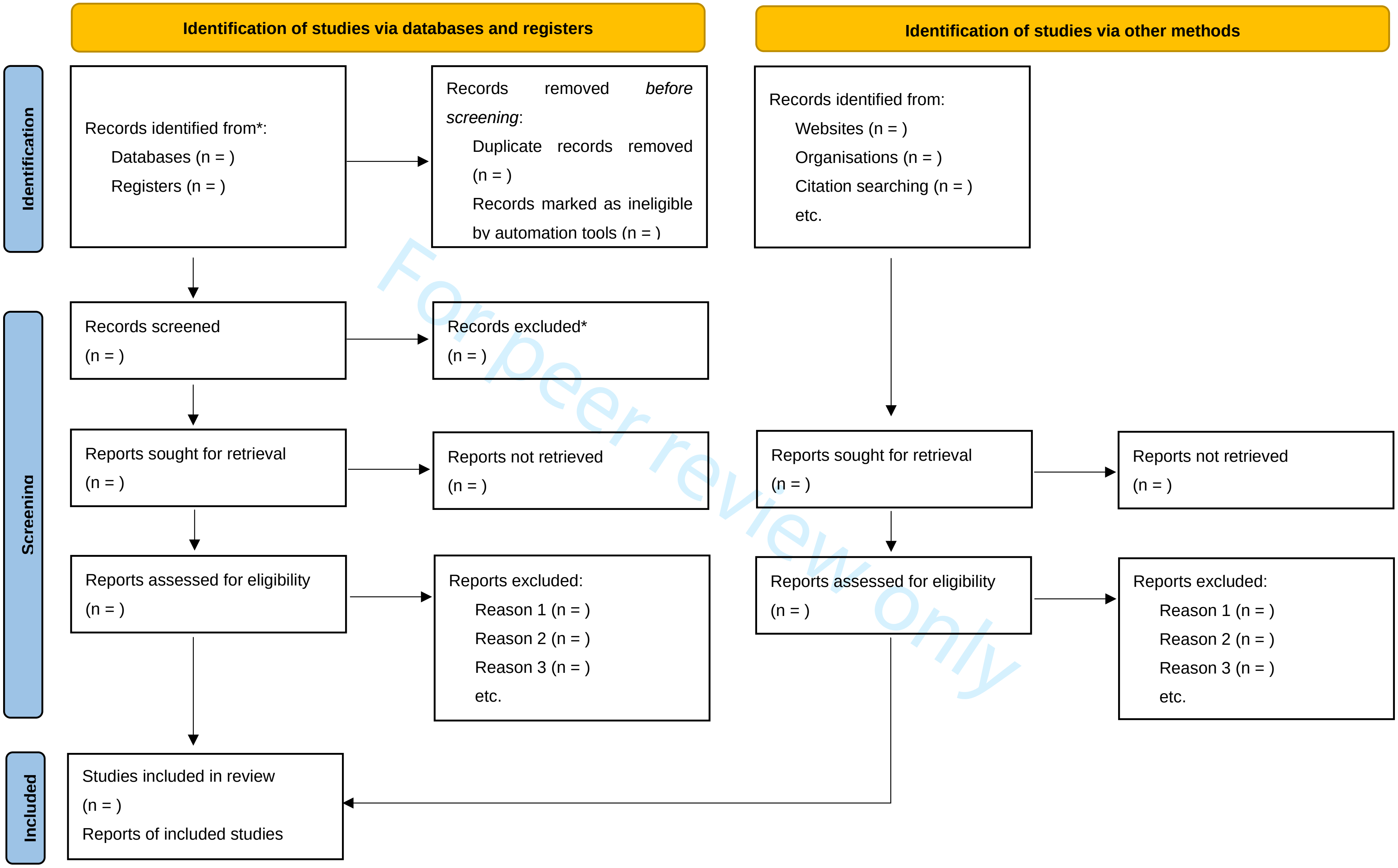

Figure1 PRISMA flow chart. *Consider, if feasible to do so, reporting the number of records identified from each database or register searched (rather than the total number across all databases/registers). **If automation tools were used, indicate how many records were excluded by a human and how many were excluded by automation tools.

PRISMA-P (Preferred Reporting Items for Systematic review and Meta-Analysis Protocols) 2015 checklist: recommended items to address in a systematic review protocol*

Section and topic	Item No	Checklist item	Pages
**ADMINISTRATIVE INFORMATION**			
Title:			
Identification	1a	Identify the report as a protocol of a systematic review	1
Update	1b	If the protocol is for an update of a previous systematic review, identify as such	
Registration	2	If registered, provide the name of the registry (such as PROSPERO) and registration number	2
Authors:			
Contact	3a	Provide name, institutional affiliation, e-mail address of all protocol authors; provide physical mailing address of corresponding author	1
Contributions	3b	Describe contributions of protocol authors and identify the guarantor of the review	13
Amendments	4	If the protocol represents an amendment of a previously completed or published protocol, identify as such and list changes; otherwise, state plan for documenting important protocol amendments	
Support:			
Sources	5a	Indicate sources of financial or other support for the review	1,13
Sponsor	5b	Provide name for the review funder and/or sponsor	1,13
Role of sponsor or funder	5c	Describe roles of funder(s), sponsor(s), and/or institution(s), if any, in developing the protocol	1,13
**INTRODUCTION**			
Rationale	6	Describe the rationale for the review in the context of what is already known	4
Objectives	7	Provide an explicit statement of the question(s) the review will address with reference to participants, interventions, comparators, and outcomes (PICO)	4
**METHODS**			
Eligibility criteria	8	Specify the study characteristics (such as PICO, study design, setting, time frame) and report characteristics (such as years considered, language, publication status) to be used as criteria for eligibility for the review	5,9.10
Information sources	9	Describe all intended information sources (such as electronic databases, contact with study authors, trial registers or other grey literature sources) with planned dates of coverage	5
Search strategy	10	Present draft of search strategy to be used for at least one electronic database, including planned limits, such that it could be repeated	5,6,7,8,9
Study records:			

For peer review only

Data management	11a	Describe the mechanism(s) that will be used to manage records and data throughout the review	10,11,12
Selection process	11b	State the process that will be used for selecting studies (such as two independent reviewers) through each phase of the review (that is, screening, eligibility and inclusion in meta-analysis)	11
Data collection process	11c	Describe planned method of extracting data from reports (such as piloting forms, done independently, in duplicate), any processes for obtaining and confirming data from investigators	11,12
Data items	12	List and define all variables for which data will be sought (such as PICO items, funding sources), any pre-planned data assumptions and simplifications	11,12
Outcomes and prioritization	13	List and define all outcomes for which data will be sought, including prioritization of main and additional outcomes, with rationale	10
Risk of bias in individual studies	14	Describe anticipated methods for assessing risk of bias of individual studies, including whether this will be done at the outcome or study level, or both; state how this information will be used in data synthesis	11,12
Data synthesis	15a	Describe criteria under which study data will be quantitatively synthesised	12
	15b	If data are appropriate for quantitative synthesis, describe planned summary measures, methods of handling data and methods of combining data from studies, including any planned exploration of consistency (such as I^2, Kendall's τ)	12
	15c	Describe any proposed additional analyses (such as sensitivity or subgroup analyses, meta-regression)	12
	15d	If quantitative synthesis is not appropriate, describe the type of summary planned	12
Meta-bias(es)	16	Specify any planned assessment of meta-bias(es) (such as publication bias across studies, selective reporting within studies)	12
Confidence in cumulative evidence	17	Describe how the strength of the body of evidence will be assessed (such as GRADE)	12

*** It is strongly recommended that this checklist be read in conjunction with the PRISMA-P Explanation and Elaboration (cite when available) for important clarification on the items. Amendments to a review protocol should be tracked and dated. The copyright for PRISMA-P (including checklist) is held by the PRISMA-P Group and is distributed under a Creative Commons Attribution Licence 4.0.**

From: Shamseer L, Moher D, Clarke M, Ghersi D, Liberati A, Petticrew M, Shekelle P, Stewart L, PRISMA-P Group. Preferred reporting items for systematic review and meta-analysis protocols (PRISMA-P) 2015: elaboration and explanation. BMJ. 2015 Jan 2;349(jan02 1):g7647.

For peer review only

BMJ Open

The safety and efficacy of intravenous or topical tranexamic acid administration in surgery: A protocol for a systematic review and network meta-analysis

Journal:	*BMJ Open*
Manuscript ID	bmjopen-2021-058093.R2
Article Type:	Protocol
Date Submitted by the Author:	19-Apr-2022
Complete List of Authors:	Wang, Xinyan; Beijing Tiantan Hospital, Anaesthesiology<br>Wang, Xinxin; Beijing Tiantan Hospital, Anaesthesiology<br>Liang, Fa; Beijing Tiantan Hospital, Anesthesiology<br>Yu, Yun; Beijing Tiantan Hospital, Anaesthesiology<br>Han, Ruquan; Beijing Tiantan Hospital,
**Primary Subject Heading**:	Surgery
Secondary Subject Heading:	Pharmacology and therapeutics, Anaesthesia, Evidence based practice, Haematology (incl blood transfusion)
Keywords:	SURGERY, Blood bank & transfusion medicine < PATHOLOGY, Thromboembolism < CARDIOLOGY

SCHOLARONE™
Manuscripts

The safety and efficacy of intravenous or topical tranexamic acid administration in surgery: A protocol for a systematic review and network meta-analysis

Xinyan Wang, MD.; Xinxin Wang, M.D.; Fa Liang, M.D.; Yun Yu, M.D.; Ruquan Han, M.D., Ph.D.

Department of Anesthesiology, Beijing Tiantan Hospital, Capital Medical University, Beijing, China

Corresponding author:

Ruquan Han, MD, PhD, Department of Anesthesiology, Beijing Tiantan Hospital, Capital Medical University, Beijing, China

Address: No. 119, Southwest 4th Ring Road, Fengtai District, Beijing, PR China, 100070

Email: ruquan.han@ccmu.edu.cn

Phone (office): 8610-59776660

Email:

Xinyan Wang: alice1428656335@126.com

Xinxin Wang: 635211696@qq.com

Liang Fa: liangfa1001@163.com

Yun Yu: yy_waitou@163.com

Ruquan Han: ruquan.han@gmail.com

Funding: This study was supported by funding from the Clinical Medicine Development of Special Funding Support from the Beijing Municipal Administration of Hospitals (ZYLX201708; DFL20180502).

The authors have no potential conflicts of interest to declare with respect to the research, authorship, and/or publication of this article.

For peer review only

ABSTRACT

Introduction Tranexamic acid (TXA) has become a widely used antifibrinolytic drug for reducing bleeding in surgery. However, adverse events, such as seizures, pulmonary embolism, and deep vein thrombosis, limit its application. To date, insufficient attention has been devoted to determining the optimal dosage and administration route of tranexamic acid in the field of surgery. Thus, this study uses the network meta-analysis method, relying on its characteristics of combining direct comparison and indirect comparison, to analyse the safety and efficacy of different doses (high, medium, low) of intravenous injection or of topical application of TXA.

Methods and analysis We will search the PubMed, Cochrane Central Register of Controlled Trials, Embase, Web of Science, and China National Knowledge Internet (CNKI) databases using a strategy that combines the terms tranexamic acid, randomized controlled trials, and embolism (or haemorrhage, blood transfusion, seizure, mortality). Two reviewers will independently screen all identified abstracts for eligibility and evaluate the risk of bias of the included studies using the Cochrane risk of bias tool for randomized controlled studies. We will conduct a systematic review and network meta-analysis. We plan to investigate heterogeneity by performing subgroup analysis and sensitivity analysis, and we will also consider the dose–response relationship between the optimal dose and a better routine. We will assess the overall certainty of the evidence for each outcome using the Grading Recommendations Assessment, Development, and Evaluation approach

Ethics and dissemination No ethics approval will be sought, as no original data will be collected for this review. Findings will be disseminated through peer-reviewed publications and conference presentations.

PROSPERO registration number CRD42021281206

Key words Tranexamic acid; Surgery; Transfusion rate; Thromboembolism

For peer review only

Strengths and limitations of this study

1. This is the first network meta-analysis to focus on the optimal dose and administration route of TXA in surgery.
2. Unpublished ongoing clinical studies will be searched using the WHO International Clinical Trials Registry Platform and ClinicalTrials.gov.
3. The quality of evidence will be assessed using the Grades Recommendations Assessment, Development, and Evaluation (GRADE)
4. We will have no language restrictions in this meta-analysis, thus yielding a more comprehensive group of eligible studies.
5. The main limitation of our study protocol is that some of the included trials may not be of high quality, which will influence our assessment of the safety and efficiency of the types of tranexamic acid administration.

For peer review only

Background

Bleeding is a major problem in surgery[1 2]. However, the common treatment—transfusion—is limited by a lack of blood sources, high processing costs, adverse blood transfusion reactions, and transmission of infectious diseases[3-5]. Recently, the concept of patient blood management (PBM) has attracted more attention[6 7]. It has been defined as the timely application of personalized, evidence-based, care bundles of interventions that reduce bleeding and transfusion to improve clinical outcomes[6-8], such as autologous blood transfusion, restricted blood transfusion, point-of-care diagnostic test-based algorithms for the personalized treatment of coagulopathy and antifibrinolytic drugs[6-8]. Among them, the antifibrinolytic drug tranexamic acid (TXA) has become widely used.

In 2021, two systematic reviews and meta-analyses of TXA were published in Annal of Surgery[2 9]. The results showed that perioperative prophylactic topical use or single-dose intravenous tranexamic acid has high safety and a low incidence of adverse events[2 9]. Nonetheless, there are still adverse events, such as seizures, pulmonary embolism, deep vein thrombosis, headache, and fatigue[10 11]. Previous research has established that postoperative seizures are associated with TXA dosage[12], which may be because of the continuous high concentration of TXA in the brain tissue at the early stage after surgery[13]. Thromboembolic events are another major concern[11 14], especially in patients who are in a hypercoagulable state due to stress during the perioperative period [15 16]. Tranexamic acid, as an antifibrinolytic drug, tends to increase coagulation function[17]. Therefore, adopting the appropriate route of administration and dosage has become the primary solution strategy.

The most popular clinical administration routes of TXA are intravenous infusion and topical use[9 17 18]. To date, far too little attention has been given to the optimal dosage and administration route of tranexamic acid in the field of surgery[19]. Conducting multidose, multiple intervention, large-scale, randomized controlled studies is time-consuming and labour-intensive. In this network meta-analysis, we plan to analyse the safety and efficacy of different doses (high, medium, low) of intravenous injection or topical application of TXA in surgical patients of all ages using direct comparison and indirect comparison[20].

For peer review only

Methods and analyses

The review has been registered in PROSPERO International Prospective Register of Systematic reviews (CRD42021281206) (https://www.crd.york.ac.uk/PROSPERO) and will be reported in accordance with the Preferred Reporting Items for Systematic reviews and Meta-Analysis protocol (PRISMA-P) guidelines[21]. The PRISMA flow diagram will be used to record every step of the review process **(Figure 1)**. We are planning to start the review in September 2021 and to complete it in December 2022 at the latest.

Sources of evidence and Search Strategy

We will include all published studies fulfilled with criteria without language restriction in this protocol. The published studies will be identified by searching the PubMed, Cochrane Central Register of Controlled Trials, Embase, Web of Science and China National Knowledge Internet (CNKI) databases from inception to September 20th, 2021 (Tables 1, 2, 3, 4, 5). Unpublished ongoing clinical studies will be identified by searching the World Health Organization International Clinical Trials Registry Platform and ClinicalTrials.gov up to September 20th, 2021. We will continue to update and include the latest articles that meet the search criteria until the deadline. The two researchers (Xinyan Wang and Xinxin Wang) will independently conduct the literature searches in accordance with the search strategy ("Tranexamic acid", "Randomized Controlled Trial" and one of the outcomes, specific strategy in Tables 1, 2, 3, 4, 5).

For peer review only

Table 1 Search strategy of PubMed

Search number	Query
#1	Tranexamic Acid [MeSH Terms]
#2	((((((((((AMCHA[Title/Abstract]) OR (trans-4-(Aminomethyl)cyclohexanecarboxylic Acid[Title/Abstract])) OR (t-AMCHA[Title/Abstract])) OR (AMCA[Title/Abstract])) OR (Anvitoff[Title/Abstract])) OR (Cyklokapron[Title/Abstract])) OR (Ugurol[Title/Abstract])) OR (KABI 2161[Title/Abstract])) OR (Spotof[Title/Abstract])) OR (Transamin[Title/Abstract])) OR (Amchafibrin[Title/Abstract])) OR (Exacyl[Title/Abstract])
#3	#1 or #2
#4	Randomized Controlled Trials as Topic [MeSH Terms]
#5	((((Clinical Trials, Randomized [Title/Abstract]) OR (Trials, Randomized Clinical [Title/Abstract])) OR (Controlled Clinical Trials, Randomized [Title/Abstract])) OR (Clinical trials [Title/Abstract])) OR (Randomized Controlled*[Title/Abstract])
#6	#4 or #5
#7	"Embolism"[MeSH Terms] OR "Embolisms"[Title/Abstract] OR "Embolus"[Title/Abstract] OR "thromboembolism"[MeSH Terms] OR "Thrombosis"[Mesh] OR "Venous Thrombosis"[Mesh]
#8	"hemorrhage"[MeSH Terms] OR "Hemorrhages"[Title/Abstract] OR "Bleeding"[Title/Abstract]
#9	"Blood transfusion"[MeSH Terms] OR "blood transfusion"[MeSH Terms] OR "blood transfusion"[MeSH Terms]
#10	"Seizures"[MeSH Terms] OR "Seizure"[Title/Abstract] OR "Mortality"[MeSH Terms] OR "Mortalities"[Title/Abstract] OR "death rate"[Title/Abstract] OR "case fatality rate"[Title/Abstract] OR "crude mortality rate"[Title/Abstract]
#11	#7 or #8 or #9 or #10
#12	#3 and #6 and #11

For peer review only

Table 2 Search strategy of the Cochrane Central Register of Controlled Trials

Search number	Query
#1	MeSH descriptor: [Tranexamic Acid] explode all trees
#2	(AMCHA or t-AMCHA or AMCA or trans-4 Aminomethyl-cyclohexane carboxylic Acid or KABI 2161 or Cyklokapron or Transamin or Spotof or Ugurol or Exacyl or Anvitoff or Amchafibrin): ti, ab, kw (Word variations have been searched)
#3	#1 or #2
#4	MeSH descriptor: [Randomized Controlled Trial] explode all trees
#5	(Randomized Control* or clinical trial*): ti, ab, kw (Word variations have been searched)
#6	#4 or #5
#7	MeSH descriptor: [Hemorrhage] explode all trees
#8	(Bleeding or Hemorrhages): ti, ab, kw (Word variations have been searched)
#9	#7 or #8
#10	MeSH descriptor: [Blood Transfusion] explode all trees
#11	("transfusion"): ti, ab, kw (Word variations have been searched)
#12	#10 or #11
#13	MeSH descriptor: [Mortality] explode all trees
#14	(Mortality Decline or Death Rate or Crude Mortality Rate): ti, ab, kw (Word variations have been searched)
#15	#13 or #14
#16	MeSH descriptor: [Thromboembolism] explode all trees
#17	MeSH descriptor: [Embolism] explode all trees
#18	(Embolisms or Embolus): ti, ab, kw (Word variations have been searched)
#19	MeSH descriptor: [Thrombosis] explode all trees
#20	MeSH descriptor: [Venous Thrombosis] explode all trees
#21	MeSH descriptor: [Seizures] explode all trees
#22	#16 or #17 or #18 or #19 or #20 or #21
#23	#9 or #12 or #15 or #22
#24	#3 and #6 and #23

For peer review only

Table 3 Search strategy of Embase

Search number	Query
#1	'Tranexamic acid'/exp
#2	((('amcha'/exp OR amcha OR 't amcha' OR 'amca'/exp OR amca OR 'trans 4') AND 'aminomethyl cyclohexane' AND carboxylic AND ('acid'/exp OR acid) OR kabi) AND 2161 OR 'cyklokapron'/exp OR cyklokapron OR 'transamin'/exp OR transamin OR spotof OR 'ugurol'/exp OR ugurol OR 'exacyl'/exp OR exacyl OR 'anvitoff'/exp OR anvitoff OR 'amchafibrin'/exp OR amchafibrin) AND [abstracts]/lim
#3	#1 OR #2
#4	'Randomized controlled trial (topic)'/exp
#5	'Clinical trial (topic)'
#6	#4 OR #5
#7	hemorrhage
#8	'bleeding'/exp
#9	#7 OR #8
#10	'Blood transfusion'/exp
#11	'transfusion'
#12	#10 OR #11
#13	'mortality'/exp
#14	((mortality AND decline OR death) AND rate OR crude) AND mortality AND rate
#15	#13 OR #14
#16	'thromboembolism'/exp
#17	'embolism'/exp
#18	embolisms OR embolus
#19	'thrombosis'/exp
#20	'Venous thrombosis'/exp
#21	#16 OR #17 OR #18 OR #19 OR #20
#22	'seizure'/exp
#23	#9 OR #12 OR #15 OR #21 OR #23
#24	#3 AND #6 AND #23

For peer review only

Table 4 Search strategy of Web of Science

Search number	Query
#1	TS= (tranexamic acid)
#2	TS= (AMCHA or (t-AMCHA) or AMCA or (trans-4-(Aminomethyl) cyclohexanecarboxylic Acid) or KABI 2161 or Cyklokapron or Transamin or Spotof or Ugurol or Exacyl or Anvitoff or Amchafibrin)
#3	#1 or #2
#4	TS= (Randomized Controlled Trial)
#5	(TS= (Randomized Control*)) OR TS= (clinical trial*)
#6	#4 or #5
#7	TS=(Hemorrhage) or AB=Bleeding or AB=Hemorrhages
#8	TS= (Blood Transfusion) or AB=transfusion
#9	TS=(Mortality) or AB=Mortality Decline or AB=Death Rate or AB=Crude Mortality Rate
#10	TS=(Thromboembolism) or TS=Embolism or AB=Embolisms or AB=Embolus
#11	TS=(Thrombosis) or TS= (Venous thrombosis)
#12	TS=(Seizure)
#13	#7 or #8 or #9 or #10 or #11 or #12
#14	#3 and #6 and #13

Table 5 Search strategy of the China National Knowledge Internet

Search number	Query
#1	检索范围；总库（摘要：氨甲环酸）AND（摘要：随机对照研究）

Inclusion and exclusion criteria

To be included in this systematic review, studies must fulfil each of the criteria outlined below.

Research type (S): randomized controlled trial (RCT).

Participant (P)

Inclusion criteria:

1) Patients (regardless of age) undergoing surgery, including orthopaedics, neurosurgery, obstetrics and gynaecology, plastic surgery, paediatrics, etc.

For peer review only

2) Intravenous or topical use of TXA, indicating the dosage.

3) Including at least one of the following outcome indicators: blood loss (intraoperative, postoperative, or total blood loss); blood transfusion; blood transfusion rate; thromboembolic events (deep vein thrombosis, pulmonary embolism), seizures, death

Exclusion criteria: Studies that did not meet the inclusion criteria, such as

1) Inconsistent research types: cohort studies, case–control studies, case reports, reviews, etc.

2) Lack of outcome indicators, lack of odds ratio (OR) and standardized mean difference (SMD) data

Intervention and groups

1) Low topical TXA, ≤1 g.

2) Medium doses of topical TXA, 1-2 g.

3) High doses of topical TXA, >2 g.

4) Intravenous low-dose TXA was defined as an infusion dose ≤1 g, an initial dose ≤10 mg/kg, or a maintenance dose ≤10 mg/kg/h.

5) Intravenous medium-dose TXA: infusion dose 1-2 g, or initial dose 10-20 mg/kg, maintenance dose≤15 mg/kg/h.

6) Intravenous high-dose TXA: infusion dose ≥2 g, initial dose >20 mg/kg, maintenance dose ≤20 mg/kg/h.

7) Placebo

Outcomes

1) Efficacy outcomes: bleeding volume, blood transfusion volume and blood transfusion rate, operation duration, postoperative Hb and Hct values, change in Hb or Hct, and drain output.

2) Safety outcomes: mortality, incidence of thromboembolism, seizures and haematoma.

Languages

There will be no language restriction. We will seek professional translators with bilingual language backgrounds for translation. For example, when we encounter an article in French, we will find a professional translator proficient in French and Chinese (or French and English) to translate the original text to ensure the accuracy of the translation.

Time

The anticipated start date is September 2021, and the anticipated completion date is December

For peer review only

2022.

Study records

Data management

The results of the literature search will be imported into the EndNote X9.3.3 database, and duplicates will be removed. We will establish several independent groups for each selecting stage in the EndNote database. Abstracts and full-text articles will be uploaded to the database. The extraction information table of the final included studies has been designed, and the study team will receive training.

Selection process

We (Xinyang Wang and Xinxiang Wang) will independently screen studies according to the inclusion and exclusion criteria. The merged results will be imported into Endnote X9.3.3, and duplicate studies will be removed.

All searched articles will be selected in a two-stage process. First, the title and abstract will be assessed based on the inclusion and exclusion criteria. The study will be removed if it does not meet the criteria. Next, full texts of articles retained in the first round of screening will be retrieved and examined based on eligibility criteria to confirm their inclusion, and studies that do not fulfil the criteria will be removed.

Both steps of the assessment will be performed independently by two reviewers (Xinyang Wang and Xinxiang Wang). If an inconsistency occurs, a third reviewer will be consulted. We will record reasons for exclusion at both stages of the inclusion process.

Data extraction

We (Xinyang Wang and Xinxiang Wang) will independently extract the following data from each study: study type, participants, inclusion criteria, exclusion criteria, baseline characteristics (age, sex, etc.), country, setting, interventions, all outcomes, findings, and study dates. After data extraction, we will compile the information and import it into Excel spreadsheets. When an inconsistency occurs, we will recheck the original document to correct the error. When dealing with missing data, we will contact principal investigators to obtain

For peer review only

unreported data or other detailed information.

Risk of bias assessment

We (Xinyang Wang and Xinxiang Wang) will evaluate the risk of bias for each included study independently. The Cochrane risk of bias tool is used to assess randomized controlled studies[22]. The evaluation scale will imported into Revman software in advance, and specific reasons will be provided for each evaluation characteristic. If an inconsistency occurs, a third reviewer (Fa Liang, Yun Yu or Ruquan Han) will be consulted.

Statistical analysis

The network meta-analysis will be performed using STATA 13.1, Revman 5.3, and R software 3.6.0. Risk ratios (RRs) with 95% confidence intervals (CIs) will be calculated using the random effects model for investigating treatment effects. A Z test will be conducted to assess the significance of the overall effect size. A P value of <0.05 will be considered statistically significant.

After constructing a heterogeneity matrix, the frequentist method will be applied to the fitted meta-regression model. The model includes covariates as the basic parameters and assumes that heterogeneity is independent of the comparison between effect sizes from multiarm studies. Inconsistency refers to the differences between direct and various indirect effects estimated for the same comparison. We will estimate the probability of a treatment being ranked at a specific place according to the outcome using "network rank."

If evidence suggests moderate statistical or clinical heterogeneity, we plan to investigate this by performing subgroup and sensitivity analyses. We will conduct subgroup and sensitivity analyses based on the actual situation of the included studies. Subgroup analysis will be performed based on sex, ethnic group, age group, sample size, type of surgery, etc., since these factors are particularly important for dose efficacy. The Instrument for assessing the Credibility of Effect Modification Analyses (ICEMAN) tool will be used to assess the credibility of subgroup analysis[23]. Sensitivity analysis will be planned without patients undergoing cardiac surgery and without paediatric patients.

For peer review only

To explore the specific value of the optimal dose and better routine, the dose–response relationship will be considered in this study to determine whether there is a threshold effect.

Publication bias will be evaluated by a "comparison-adjusted" funnel plot. GRADEpro software will be used to grade the evidence of all the outcomes, and this process will be completed by two individuals separately.

Patient and public involvement: This study protocol did not involve either patients or the public.

Amendments: If there are any amendments to the protocol, we will explain in the final report.

Ethics and Dissemination: No ethics approval will be sought, as no original data will be collected for this review. Findings will be disseminated through peer-reviewed publication and conference presentations.

Contributors: Xinyan Wang: study design, conduct of study, bibliographic research, design of data entry forms, data management, protocol and manuscript writing and review. Xinxin Wang: bibliographic research design and conduct, protocol, and manuscript review. Liang Fa: protocol and manuscript review. Yun Yu: protocol and manuscript review. Ruquan Han: study conception and design, scientific coordination, protocol, and manuscript writing and review.

Funding: This study was supported by funding from the Clinical Medicine Development of Special Funding Support from the Beijing Municipal Administration of Hospitals (ZYLX201708; DFL20180502).

Competing interests: None declared.

Patient consent for publication: Not required.

Provenance and peer review: Not commissioned; externally peer reviewed.

Open access: This is an open access article distributed in accordance with the Creative Commons Attribution Non-Commercial (CC BY-NC 4.0) licence, which permits others to distribute, remix, adapt, build upon this work noncommercially, and licence their derivative works on different terms, provided the original work is properly cited, appropriate credit is given, any changes made indicated, and the use is noncommercial. See:

For peer review only

http://creativecommons.org/licenses/by-nc/4.0/.

For peer review only

REFERENCES

1. Rasouli MR, Maltenfort MG, Erkocak OF, et al. Blood management after total joint arthroplasty in the United States: 19-year trend analysis. *Transfusion* 2016;56(5):1112-20.
2. Teoh WY, Tan TG, Ng KT, et al. Prophylactic Topical Tranexamic Acid Versus Placebo in Surgical Patients: A Systematic Review and Meta-analysis*. *Ann Surg* 2021;273(4):676-83.
3. Alter HJ, Klein HG. The hazards of blood transfusion in historical perspective. *Blood* 2008;112(7):2617-26. doi: 10.1182/blood-2008-07-077370 [published Online First: 2008/09/24]
4. Spahn DR, Goodnough LT. Alternatives to blood transfusion. *Lancet* 2013;381(9880):1855-65.
5. Stokes EA, Wordsworth S, Staves J, et al. Accurate costs of blood transfusion: a microcosting of administering blood products in the United Kingdom National Health Service. *Transfusion* 2018;58(4):846-53.
6. Spahn DR, Muñoz M, Klein AA, et al. Patient Blood Management: Effectiveness and Future Potential. *Anesthesiology* 2020;133(1):212-22.
7. Roman MA, Abbasciano RG, Pathak S, et al. Patient blood management interventions do not lead to important clinical benefits or cost-effectiveness for major surgery: a network meta-analysis. *Br J Anaesth* 2021;126(1):149-56.
8. Mueller MM, Van Remoortel H, Meybohm P, et al. Patient Blood Management: Recommendations From the 2018 Frankfurt Consensus Conference. *Jama* 2019;321(10):983-97.
9. Heyns M, Knight P, Steve AK, et al. A Single Preoperative Dose of Tranexamic Acid Reduces Perioperative Blood Loss: A Meta-analysis. *Ann Surg* 2021;273(1):75-81.
10. Chauncey JM, Wieters JS: Tranexamic Acid. In *StatPearls*. edn.Treasure Island (FL): StatPearls Publishing Copyright © 2021, StatPearls Publishing LLC. 2021.
11. Murao S, Nakata H, Yamakawa K. Safety of tranexamic acid in thrombotic adverse events and seizure in patients with haemorrhage: a protocol for a systematic review and meta-analysis. *BMJ Open* 2020;10(6):e036020.
12. Kalavrouziotis D, Voisine P, Mohammadi S, et al. High-dose tranexamic acid is an independent predictor of early seizure after cardiopulmonary bypass. *Ann Thorac Surg* 2012;93(1):148-54.
13. Lecker I, Wang DS, Whissell PD, et al. Tranexamic acid-associated seizures: Causes and treatment. *Ann Neurol* 2016;79(1):18-26.
14. Poeran J, Chan JJ, Zubizarreta N, et al. Safety of Tranexamic Acid in Hip and Knee Arthroplasty in High-risk Patients. *Anesthesiology* 2021;135(1):57-68.
15. Wu H, Wang Y, Zhang Y, et al. Breaking the vicious loop between inflammation, oxidative stress and coagulation, a novel anti-thrombus insight of nattokinase by inhibiting LPS-induced inflammation and oxidative stress. *Redox Biol* 2020;32:101500.
16. Senoner T, Schindler S, Stättner S, et al. Associations of Oxidative Stress and Postoperative Outcome in Liver Surgery with an Outlook to Future Potential Therapeutic Options. *Oxid Med Cell Longev* 2019;2019:3950818.
17. Xu S, Chen JY, Zheng Q, et al. The safest and most efficacious route of tranexamic acid

For peer review only

administration in total joint arthroplasty: A systematic review and network meta-analysis. *Thromb Res* 2019;176:61-66.

18. Wang X, Yang R, Sun H, et al. Different Effects of Intravenous, Topical, and Combined Application of Tranexamic Acid on Patients with Thoracolumbar Fracture. *World Neurosurg* 2019;127:e1185-e89.
19. Fillingham YA, Ramkumar DB, Jevsevar DS, et al. The Efficacy of Tranexamic Acid in Total Hip Arthroplasty: A Network Meta-analysis. *J Arthroplasty* 2018;33(10):3083-89.e4.
20. Rouse B, Chaimani A, Li T. Network meta-analysis: an introduction for clinicians. *Intern Emerg Med* 2017;12(1):103-11.
21. Shamseer L, Moher D, Clarke M, et al. Preferred reporting items for systematic review and meta-analysis protocols (PRISMA-P) 2015: elaboration and explanation. *Bmj* 2015;350:g7647.
22. Higgins JP, Altman DG, Gøtzsche PC, et al. The Cochrane Collaboration's tool for assessing risk of bias in randomised trials. *Bmj* 2011;343:d5928.
23. Schandelmaier S, Briel M, Varadhan R, et al. Development of the Instrument to assess the Credibility of Effect Modification Analyses (ICEMAN) in randomized controlled trials and meta-analyses. *Cmaj* 2020;192(32):E901-e06.

For peer review only

Figure and table legends

Table 1. Search Strategy for PubMed

Table 2. Search Strategy for the Cochrane Central Register of Controlled Trials

Table 3. Search Strategy for Embase

Table 4. Search Strategy for Web of Science

Table 5. Search Strategy for the China National Knowledge Internet

Figure 1. Flow chart diagram presents the selection of articles for systemic review and network meta-analysis of the safety and efficacy of intravenous or topical tranexamic acid administration in surgery.

For peer review only

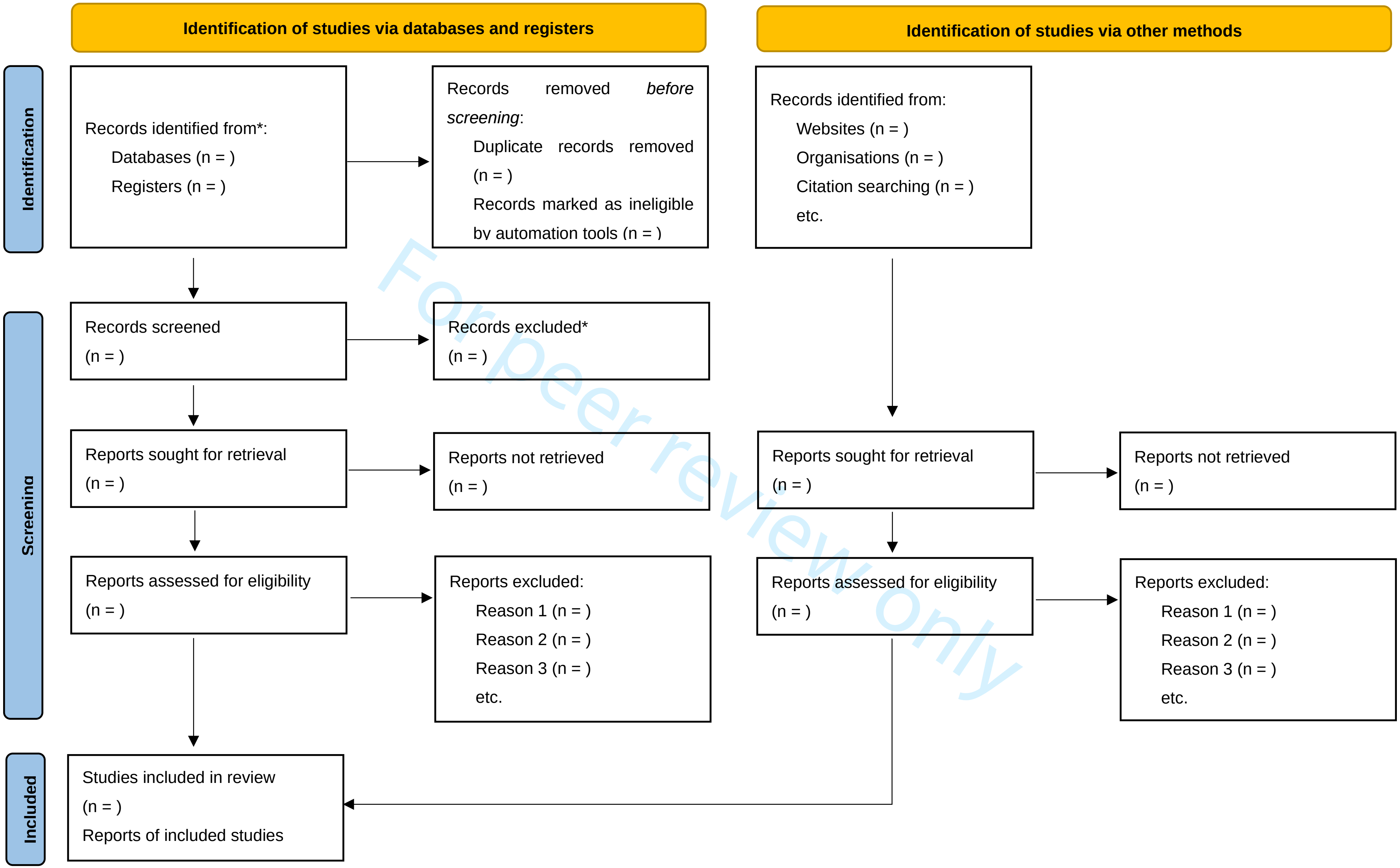

For peer review only

Figure1 PRISMA flow chart. *Consider, if feasible to do so, reporting the number of records identified from each database or register searched (rather than the total number across all databases/registers). **If automation tools were used, indicate how many records were excluded by a human and how many were excluded by automation tools.

For peer review only - http://bmjopen.bmj.com/site/about/guidelines.xhtml

PRISMA-P (Preferred Reporting Items for Systematic review and Meta-Analysis Protocols) 2015 checklist: recommended items to address in a systematic review protocol*

Section and topic	Item No	Checklist item	Pages
**ADMINISTRATIVE INFORMATION**			
Title:			
Identification	1a	Identify the report as a protocol of a systematic review	1
Update	1b	If the protocol is for an update of a previous systematic review, identify as such	
Registration	2	If registered, provide the name of the registry (such as PROSPERO) and registration number	2
Authors:			
Contact	3a	Provide name, institutional affiliation, e-mail address of all protocol authors; provide physical mailing address of corresponding author	1
Contributions	3b	Describe contributions of protocol authors and identify the guarantor of the review	13
Amendments	4	If the protocol represents an amendment of a previously completed or published protocol, identify as such and list changes; otherwise, state plan for documenting important protocol amendments	
Support:			
Sources	5a	Indicate sources of financial or other support for the review	1,13
Sponsor	5b	Provide name for the review funder and/or sponsor	1,13
Role of sponsor or funder	5c	Describe roles of funder(s), sponsor(s), and/or institution(s), if any, in developing the protocol	1,13
**INTRODUCTION**			
Rationale	6	Describe the rationale for the review in the context of what is already known	4
Objectives	7	Provide an explicit statement of the question(s) the review will address with reference to participants, interventions, comparators, and outcomes (PICO)	4
**METHODS**			
Eligibility criteria	8	Specify the study characteristics (such as PICO, study design, setting, time frame) and report characteristics (such as years considered, language, publication status) to be used as criteria for eligibility for the review	5,9.10
Information sources	9	Describe all intended information sources (such as electronic databases, contact with study authors, trial registers or other grey literature sources) with planned dates of coverage	5
Search strategy	10	Present draft of search strategy to be used for at least one electronic database, including planned limits, such that it could be repeated	5,6,7,8,9
Study records:			

For peer review only

Data management	11a	Describe the mechanism(s) that will be used to manage records and data throughout the review	10,11,12
Selection process	11b	State the process that will be used for selecting studies (such as two independent reviewers) through each phase of the review (that is, screening, eligibility and inclusion in meta-analysis)	11
Data collection process	11c	Describe planned method of extracting data from reports (such as piloting forms, done independently, in duplicate), any processes for obtaining and confirming data from investigators	11,12
Data items	12	List and define all variables for which data will be sought (such as PICO items, funding sources), any pre-planned data assumptions and simplifications	11,12
Outcomes and prioritization	13	List and define all outcomes for which data will be sought, including prioritization of main and additional outcomes, with rationale	10
Risk of bias in individual studies	14	Describe anticipated methods for assessing risk of bias of individual studies, including whether this will be done at the outcome or study level, or both; state how this information will be used in data synthesis	11,12
Data synthesis	15a	Describe criteria under which study data will be quantitatively synthesised	12
	15b	If data are appropriate for quantitative synthesis, describe planned summary measures, methods of handling data and methods of combining data from studies, including any planned exploration of consistency (such as I^2, Kendall's τ)	12
	15c	Describe any proposed additional analyses (such as sensitivity or subgroup analyses, meta-regression)	12
	15d	If quantitative synthesis is not appropriate, describe the type of summary planned	12
Meta-bias(es)	16	Specify any planned assessment of meta-bias(es) (such as publication bias across studies, selective reporting within studies)	12
Confidence in cumulative evidence	17	Describe how the strength of the body of evidence will be assessed (such as GRADE)	12

*** It is strongly recommended that this checklist be read in conjunction with the PRISMA-P Explanation and Elaboration (cite when available) for important clarification on the items. Amendments to a review protocol should be tracked and dated. The copyright for PRISMA-P (including checklist) is held by the PRISMA-P Group and is distributed under a Creative Commons Attribution Licence 4.0.**

From: Shamseer L, Moher D, Clarke M, Ghersi D, Liberati A, Petticrew M, Shekelle P, Stewart L, PRISMA-P Group. Preferred reporting items for systematic review and meta-analysis protocols (PRISMA-P) 2015: elaboration and explanation. BMJ. 2015 Jan 2;349(jan02 1):g7647.

For peer review only
